# Intravital imaging reveals improved Kupffer cell-mediated phagocytosis as a mode of action of glycoengineered anti-CD20 antibodies

**DOI:** 10.1038/srep34382

**Published:** 2016-10-04

**Authors:** Capucine L. Grandjean, Fabricio Montalvao, Susanna Celli, David Michonneau, Beatrice Breart, Zacarias Garcia, Mario Perro, Olivier Freytag, Christian A. Gerdes, Philippe Bousso

**Affiliations:** 1 Dynamics of Immune Responses Unit, Equipe Labéllisée Ligue Contre le Cancer, Institut Pasteur, 75015 Paris, France; 2 INSERM U1223, 75015 Paris, France; 3 Roche Innovation Center Zurich, Roche Pharma Research & Early Development, Wagistrasse 18,8952 Schlieren, Switzerland

## Abstract

Anti-CD20 monoclonal antibodies (mAbs) represent an effective treatment for a number of B cell malignancies and autoimmune disorders. Glycoengineering of anti-CD20mAb may contribute to increased anti-tumor efficacy through enhanced antibody-dependent cellular cytotoxicity (ADCC) and phagocytosis (ADP) as reported by *in vitro* studies. However, where and how glycoengineered Ab may potentiate therapeutic responses *in vivo* is yet to be elucidated. Here, we have performed mouse liver transplants to demonstrate that the liver is sufficient to mediate systemic B cells depletion after anti-CD20 treatment. Relying on intravital two-photon imaging of human CD20-expressing mice, we provide evidence that ADP by Kupffer cells (KC) is a major mechanism for rituximab-mediated B cell depletion. Notably, a glycoengineered anti-mouse CD20 Ab but not its wild-type counterpart triggered potent KC-mediated B cell depletion at low doses. Finally, distinct thresholds for KC phagocytosis were also observed for GA101 (obinutuzumab), a humanized glycoengineered type II anti-CD20 Ab and rituximab. Thus, we propose that enhanced phagocytosis of circulating B cells by KC represents an important *in vivo* mechanism underlying the improved activity of glycoengineered anti-CD20 mAbs.

Anti-CD20 Ab is an effective therapy to treat B cell malignancies and a series of autoimmune diseases^1^,^2^,^3^. Dissecting its mode of action remains essential for the rational design of improved antibodies. Several *in vitro* studies have contributed to the delineation of distinct possible mechanisms of action^4^ but few reports have examined their respective contribution *in vivo*. We recently visualized the role of Kupffer cells in mediating B cell phagocytosis within minutes of injection of a depleting mouse anti-mouse CD20 Ab^5^. In line with this, Kupffer cells were also implicated in the clearance of circulating B16 tumor cells following administration of a tumor-specific mAb^6^. Thus, B cell recirculation^7^ combined with depletion in the liver may contribute to the systemic elimination of the B cell compartment. However, whether this mechanism is relevant for other anti-CD20 Abs and whether the liver is sufficient by itself to induce a broad B cell depletion remains to be established. Moreover, it remains unclear whether Kupffer-mediated B cell depletion can be tuned and improved by designing anti-CD20 Abs with increased potency. In particular, glycoengineering of anti-CD20 Fc portions has been shown to improve antibody-dependent cellular cytotoxicity by NK cells and to increase phagocytosis by macrophages *in vitro*^8^,^9^,^10^,^11^. To extend our previous findings, here we use both orthotopic liver transplants^12^ and intravital imaging^13^,^14^ to assess the importance of Kupffer cell-mediated B cell phagocytosis for the activity of mouse and human anti-CD20 Abs. Specifically, we address the contribution of anti-CD20 Ab glycoengineering to the efficacy of B cell depletion in the liver.

## Methods

### Liver transplant

Orthotopic liver transplantation was adapted from Kamada *et al*.^12^ and performed under a microdissecting microscope. To prepare mouse donor livers, ligaments, esophageal, pyloric, right adrenal veins and the hepatic artery were cut. Liver were perfused and harvested after cutting the inferior vena cava, the portal vein, and the superior vena cava. The inferior vena cava and the portal vein were prepared using the ‘cuff technique’. For mouse recipient liver removal, sutures were placed on the portal vein and the superior vena cava, the portal vein, and the inferior vena cava were clumped before being cut. Anastomoses were performed by suture or by sliding recipient vessels over cuffed donor vessels.

### Mice and antibodies

Wild type C57BL/6 mice were purchased from Charles River France. hCD20Tg mice^7^ (Genentech, USA), C57BL/6 GFP^+^ and FcRγ^−/−^ mice were bred in our facility. All experiments were carried out in agreement with relevant guidelines and regulations and approved by the Institut Pasteur committee on Animal Welfare (CETEA) under the protocol code of CETEA2013–0077. Anti-CD20 mAbs included 5D2 (mIgG2a, Genentech), WT and GE 18B12 (mIgG2a, Roche), rituximab and GA101 (Hôpital Necker), HY1.2 (mIgG2a) isotype control and mg053 (hIgG1) isotype control. 18B12 Ab glycoengineering was conducted using the GlycoMab technology (Roche Glycart AG)^15^.

### Immunofluorescence

Perfused livers were cut, fixed overnight in paraformaldehyde and progressively dehydrated in sucrose. Tissues were snap frozen in OCT compound (Tissue-Tek; Sakura). Sections were stained with anti-F4/80 and anti-B220 Abs.

### Intravital two-photon imaging

Intravital imaging of the liver was performed using an upright DM 6000B/SP5 microscope (Leica Microsystems) and Chameleon Ultra Ti:Sapphire laser (Coherent) tuned at 960 nm. The liver was exposed and a microscope slide was inserted underneath, immobilized using silicone paste (Express 2 Putty Quick, 3M-Espe). A coversplit was placed on the top of the liver maintained by silicone paste.

## Results and Discussion

**Figure 1:**
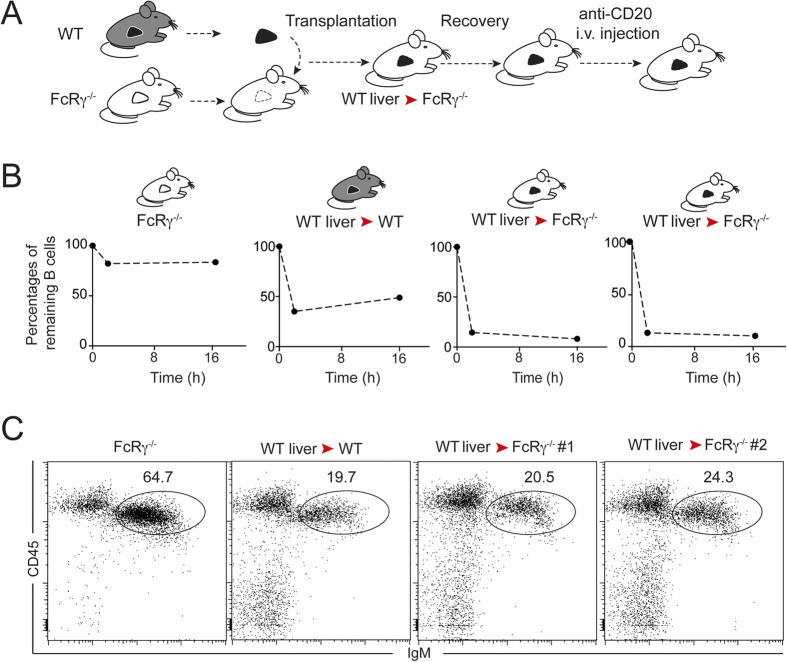
The liver is sufficient to mediate systemic B cell depletion following anti-CD20 treatment. (**A**) Experimental set-up. A surgical procedure was undertaken to transplant WT livers into two FcRγ^−/−^ and one WT recipients. Mice were allowed to recover for 3 to 7 days before being injected with a single dose (50 μg) of anti-CD20 Ab (5D2). Non-transplanted FcRγ^−/−^ mice were used as negative controls. (**B**) B cell frequency measured by flow cytometry in blood before, 2 h and 16 h following anti-CD20 treatment in the indicated mice. Values were normalized to the frequency measured prior to treatment. (**C**) B cell frequency in the spleen of WT and FcRγ^−/−^recipients transplanted with a WT liver was measured by flow cytometry at 16 hr post-treatment. Numbers show the percentage of B cells after treatment.

**Figure 2:**
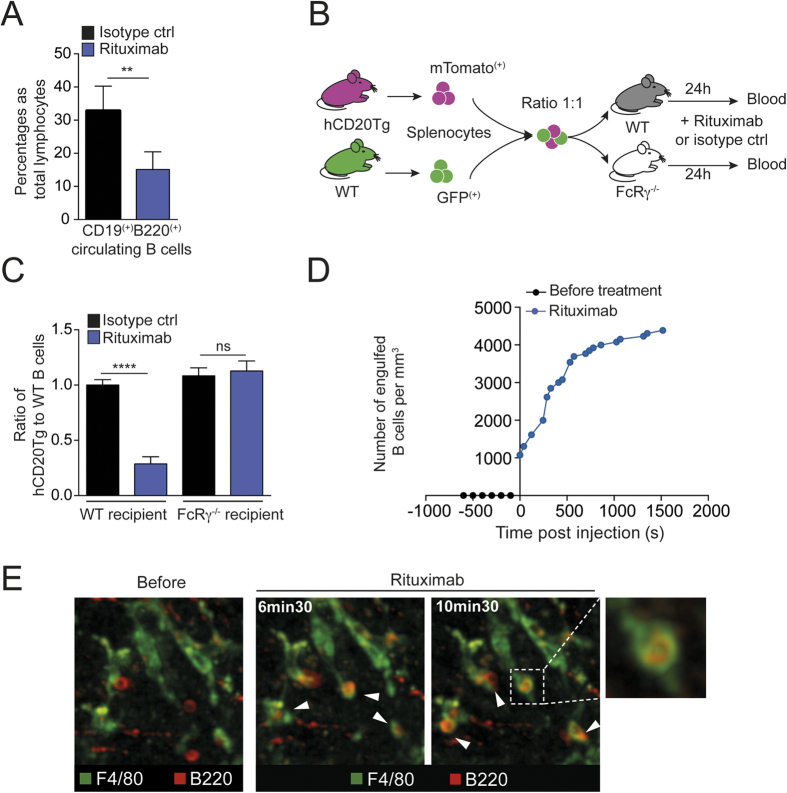
B cell depletion upon rituximab injection is mediated by Kupffer cells in human CD20 transgenic mice. (**A**) Summary bar charts of the frequency of B cells in the blood of hCD20Tg mice 5 h following injection of 50 μg rituximab or isotype control. (**B**) Experimental set-up. Splenocytes from WT (GFP^+^) or hCD20Tg (mTomato^+^) mice were isolated and co-transferred into WT or FcRγ^−/−^ recipient animals. After 24 h, mice were treated i.v. with 50 μg rituximab or isotype control. (**C**) Summary bar charts of the ratio of hCD20Tg to WT B cells in the blood 24 h following i.v. injection of 50 μg rituximab or isotype control. (**D**,**E**) hCD20Tg mice were subjected to intravital imaging of the liver. Kupffer cells (green) and B cells (red) were labeled by i.v. injection of fluorescently labeled anti-F4/80 Ab (2 μg) and anti-B220 Fab fragments, respectively. (**D**) Representative curve showing the number of engulfed B cells (normalized per mm^3^) in the liver before and after rituximab (200 μg) treatment. (**E**) Representative two-photon images obtained before and after rituximab treatment (200 μg), highlighting rapid B cell phagocytosis by Kupffer cells (white arrows). Representative of 2–4 independent experiments. Results are shown as mean ± SEM. Significance was assessed using an unpaired Student t-test.

We have previously shown using partial hepatectomy that the liver is necessary for optimal B cell depletion by a mouse anti-CD20 Ab (5D2)^5^, a mechanism shown to be dependent on activating FcRs^5^,^16^. In addition, we and others have shown that splenectomy has little effect on anti-CD20-mediated B cell depletion^5^,^7^. To assess whether the liver is in fact sufficient to mediate systemic B cell depletion, we sought to create a system in which Fc-dependent effector functions are restricted to the liver. For this purpose, we relied on orthotopic liver transplantation, a particularly challenging surgical procedure in mice due to their small size (Fig. 1A). Nevertheless, we successfully grafted two FcRγ^−/−^ recipients with a WT liver and as a positive control, a WT recipient grafted with a WT liver. Transplanted mice were treated with anti-CD20 Ab. PBL were collected prior to injection, at 2 h and 16 h post-injection while spleens were harvested at 16 h. B cell depletion occurred in the WT transplanted control following treatment, confirming that the graft procedure had no advert effects on the depletion process. Most importantly, B cells were efficiently depleted in both grafted FcRγ^−/−^ recipients carrying a WT liver, as measured in blood (Fig. 1B) and spleens (Fig. 1C) indicating that the liver is indeed sufficient to mediate broad B cell depletion of peripheral B cells. As expected^5^,^16^, no depletion was observed in non-transplanted FcRγ^−/−^ mice. We subsequently interrogated whether our previous findings using a murine anti-mouse CD20Ab also pertain to clinically relevant anti-human CD20Abs such as rituximab. Treatment of BAC transgenic mice expressing human CD20 (hCD20Tg)^7^ showed rapid B cell systemic depletion after a single injection of rituximab (Fig. 2A and Fig. S2) consistent with a previous study^17^ and this effect was FcγR-dependent (Fig. 2B,C). Intravital two-photon imaging of hCD20Tg liver revealed that circulating B cells arrested in the liver next to Kupffer cells and were engulfed within minutes of rituximab injection. Moreover, within 20 min, most B cells had been phagocytized with only very few circulating B cells being detected in the liver at this time (Fig. 2D,E, Movie S1). Taken together, these data further highlight the importance of the liver as a major B cell depletion site in response to both anti-mouse or anti-human CD20 Abs.

**Figure 3:**
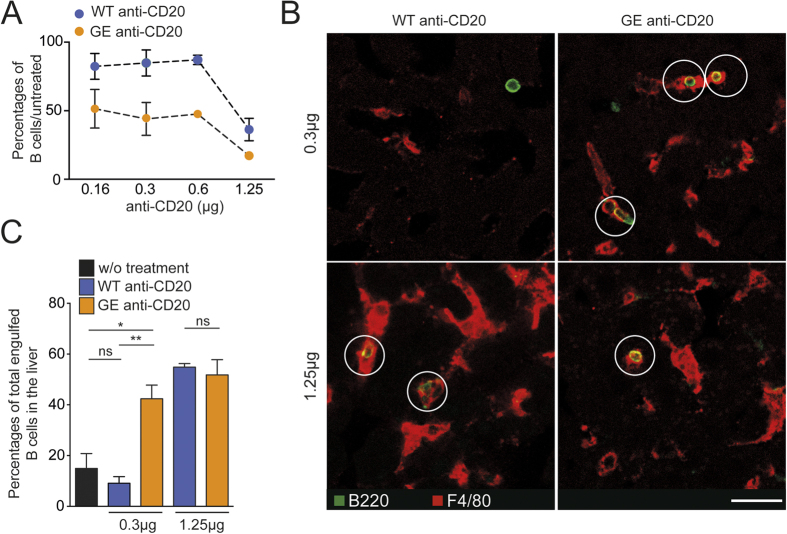
Mouse glycoengineered anti-CD20 Abs trigger enhanced Ab-dependent phagocytosis by Kupffer cells *in vivo*. **A–C**) WT or glycoengineered anti-CD20 mAbs (clone18B12) were used to treat WT mice at the indicated doses. (**A**) B cell depletion efficacy was assessed in blood by flow cytometry at 30 min post-injection. (**B**) Frozen liver sections were stained using PE-labeled anti-F4/80 Ab and FITC-labeled anti-B220 Ab. B cell phagocytosis by Kupffer cells is highlighted by white circles. Note that only the glycoengineered form of anti-CD20 trigger phagocytosis at low doses. (**C**) Summary bar charts showing percentages of engulfed B cells for the indicated conditions. Representative of 2–4 independent experiments. Results are shown as mean ± SEM and were compiled from mosaic images of liver sections (3 independent animals) containing > 1000 Kupffer cells.

**Figure 4:**
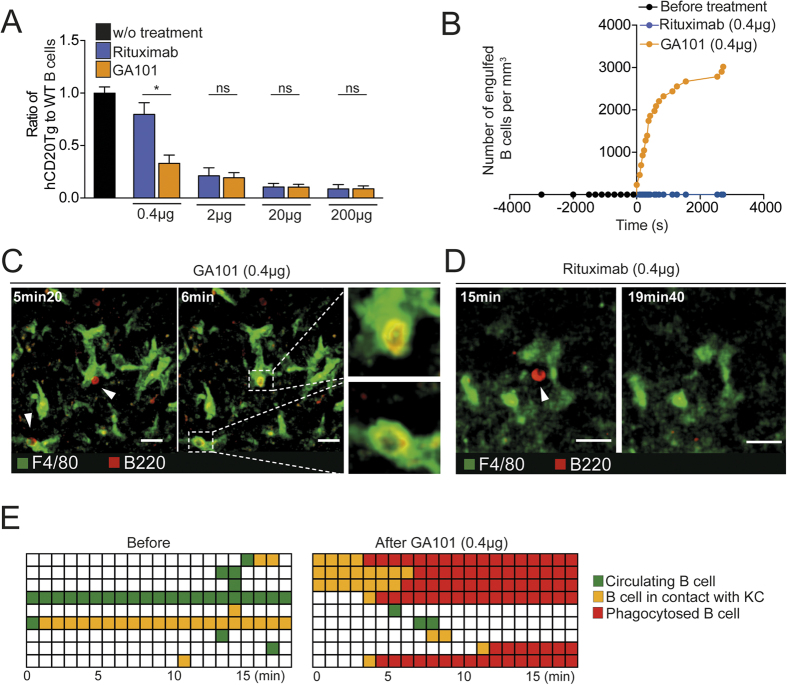
Obinutuzumab triggers enhanced Ab-dependent phagocytosis by Kupffer cells *in vivo* compared to rituximab. (**A**) Splenocytes from WT or hCD20Tg mice were isolated, labeled and co-transferred into WT recipient mice. After 24 h, mice were treated i.v. with different doses of rituximab or GA101 and blood was analyzed 1 hr later by flow cytometry. The summary bar charts show the ratio of hCD20Tg to WT B cells (non depleted, used as an internal control) 1 h after injection of the indicated dose of rituximab or GA101. (**B–E**) Intravital imaging of the liver of hCD20Tg mice during anti-CD20 treatment. Kupffer cells (green) and B cells (red) were labeled using anti-F4/80 Ab and anti-B220 Fab fragments, respectively. (**B**) Representative curve showing the number of engulfed B cells (normalized per mm^3^) in the liver following 0.4 μg GA101. (**C**) Figure shows representative two-photon images before and after treatment with low doses of GA101 (0.4 μg), highlighting efficient B cell phagocytosis by Kupffer cells (white squares and insets). Scale bar, 25 μm. (**D**) Representative two-photon images highlighting the absence of B cell phagocytosis following 0.4 μg rituximab. Scale bar, 20 μm. (**E**) Each line represents the cell behavior after anti-CD20 injection. Green squares represent cicrculating B cells, yellow squares represent contact between a B and Kupffer cell and red squares represent engulfed B cells. Representative of 2–4 independent experiments. Results are shown as mean ± SEM. Significance was assessed using an unpaired Student t-test.

Based on these results, we investigated whether modifications to anti-CD20 Abs could tune Kupffer-cell mediated B cell phagocytosis efficacy. In this respect, glycoengineered anti-CD20 Abs have been shown to display higher potency for ADCC and APD *in vitro*^11^,^18^, but how these differences may translate *in vivo* remain to be ascertained. First, a murine anti-mouse CD20 Ab (clone 18B12, referred to as WT anti-CD20) and its glycoenginnered counterpart (GE anti-CD20) were compared for their B cell depletion efficacy *in vivo* at 30 min post-injection. Flow cytometric analyses revealed that early B cell depletion was more efficient with GE anti-CD20 compared to WT anti-CD20 particularly at low doses (Fig. 3A). Quantification of engulfed B cells in liver tissue sections identified a lower triggering threshold for GE anti-CD20 Ab (being active at doses as low as 0.3μg) compared to WT anti-CD20 Ab (Fig. 3B,C Fig. S3), a finding that was also confirmed by intravital imaging (Movie S2). Finally, using hCD20Tg mice, we compared two clinically relevant anti-human CD20 Abs, namely rituximab and obinutuzumab (GA101), for their capacity to trigger Kupffer cell-mediated B cell phagocytosis *in vivo*. GA101 is a glycoengineered type II anti-CD20 Ab shown to induce robust ADCC and APD *in vitro*^11^,^18^ due to the low fucose content of its Fc-portion, a feature that may contribute to its preclinical^19^ and clinical activity^20^. Consistently, we found a more potent B cell depletion by GA101 at low doses (Fig. 4A). To investigate how Kupffer cell activity may contribute to these differences, we relied on two-photon liver imaging of hCD20Tg mice in response to rituximab and GA101. Interestingly, we visualized very efficient B cell arrest in the liver and subsequent phagocytosis by Kupffer cells in response to low doses of GA101 whereas B cell continued to rapidly circulate in liver sinusoids of mice injected with the same dose of rituximab (Fig. 4B–E, Movie S3 and 4). Thus, distinct features of anti-CD20 Ab such as glycoengineering, but also potentially epitope specificity, type I versus type II or ability to induce antigenic modulation^17^,^21^ may confer variable Kupffer cell-mediated depletion activity during therapy.

In summary, we have used five different mAbs directed against the murine or the human CD20 molecule to show that antibody-dependent phagocytosis by Kupffer cells is a general mechanism for the systemic depletion of circulating B cells. In addition, we provide evidence that the improved potency of glycoengineered anti-CD20 Abs in mediating B cell depletion *in vivo* is linked to their enhanced capacity to trigger Kupffer cell-mediated B cell arrest and subsequent phagocytosis. Future work could address whether additional mechanisms contribute to the elimination of non-circulating malignant B cells. Intravital imaging may help optimize mAbs therapy by assessing how specific Ab modifications may finely tune their mode of action *in vivo*.

## Additional Information

**How to cite this article**: Grandjean, C. L. *et al*. Intravital imaging reveals improved Kupffer cell-mediated phagocytosis as a mode of action of glycoengineered anti-CD20 antibodies. *Sci. Rep*. **6**, 34382; doi: 10.1038/srep34382 (2016).

## Supplementary Material

Supplementary Information

Supplementary Movie S1

Supplementary Movie S2

Supplementary Movie S3

Supplementary Movie S4
