# Supplementary material for: Intravital imaging reveals improved Kupffer cell-mediated phagocytosis as a mode of action of glycoengineered anti-CD20 antibodies
Source: Sci Rep. 2016 Oct 4;6:34382. doi: 10.1038/srep34382 (PMC5048169; doi:10.1038/srep34382)
Supplement: Supplementary Information [file srep34382-s1.pdf]

# Intravital imaging reveals improved Kupffer cell-mediated phagocytosis as a mode of action of glycoengineered anti-CD20 antibodies

Capucine L. Grandjean, Fabricio Montalvao, Susanna Celli, David Michonneau,

Beatrice Breart, Zacarias Garcia, Mario Perro,

Olivier Freytag, Christian A. Gerdes and Philippe Bousso

## Supplemental materials

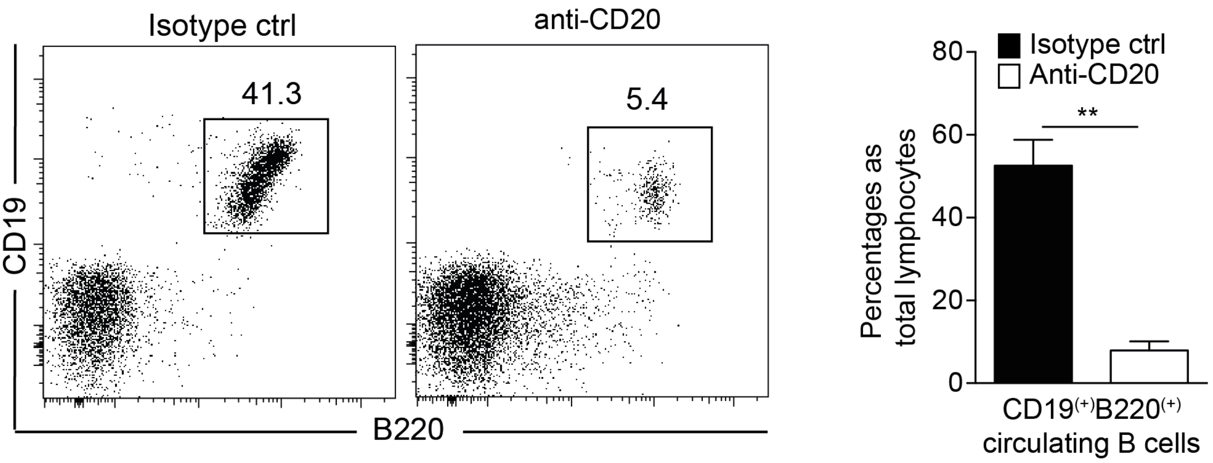


**Supplementary Figure 1. Efficient B cell depletion by the 5D2 mouse anti-mouse CD20 Ab.** WT mice were treated with 50µg of anti-CD20 (5D2) or isotype control (HY1.2) for 24h. Summary bar charts showing percentages of B cells in the blood of treated mice.

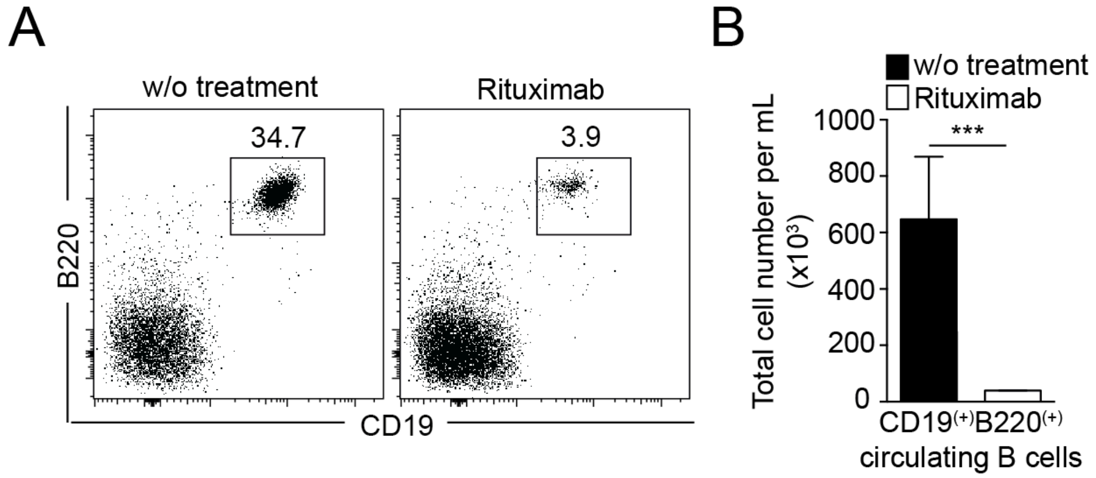


**Supplementary Figure 2. Rituximab efficiently depletes B cells in hCD20Tg mice**

hCD20Tg mice were treated with 200µg Rituximab for 5hrs or left untreated. **A**) Representative flow cytometry profiles of B cells in blood. B) Summary bar charts showing B cells/ml of blood in untreated or Rituximab treated mice.

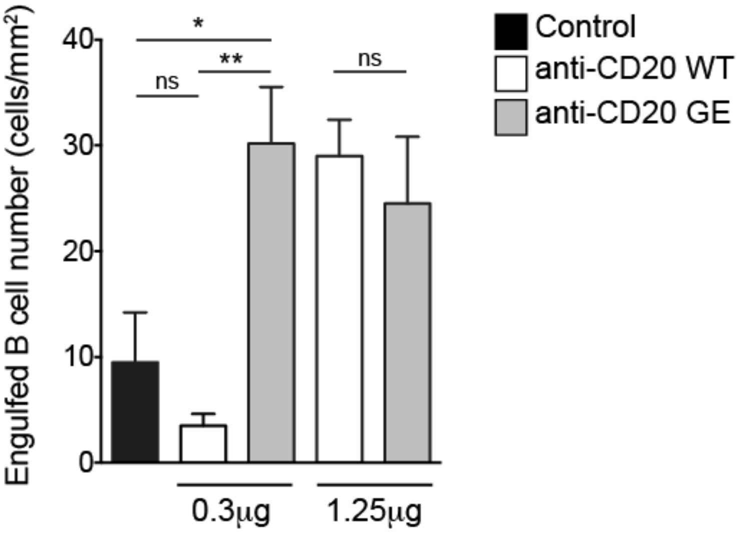


**Supplementary Figure 3. Glycoengineered anti-CD20 Ab (18B12) triggers enhanced Ab-dependent phagocytosis by Kupffer cells *in vivo.***

WT or glycoengineered anti-CD20 mAbs (clone18B12) were used to treat WT mice at the indicated doses. Frozen liver sections were stained using PE-labeled anti-F4/80 Ab and FITC-labeled anti-B220 Ab. Summary bar charts showing the density of engulfed B cells for the indicated conditions.

## Movie legends

**Movie S1. Intravital two-photon imaging of Kupffer cell-mediated B cell phagocytosis following rituximab injection.**

hCD20Tg mice were injected i.v. with 0.2µg F4/80-FITC and B220-PE to visualize Kupffer and B cells respectively. Mice were prepared for intravital liver imaging and subsequently treated with 200µg rituximab.

**Movie S2. Efficient Kupffer cell-mediated B cell phagocytosis triggered by low doses of glycoengineered anti-CD20 Ab.**

MAFIAxCD19$^{cre}$xRosaRFPxCFP mice were used to visualize B cell (red) phagocytosis by Kupffer cells (green) following 0.3µg GE anti-CD20 injection. The liver architecture appears in blue.

**Movie S3. Low doses of GA101 trigger robust Kupffer cell-mediated B cell phagocytosis**

hCD20Tg mice were injected i.v. with 2µg FITC-conjugated anti-F4/80 Ab and B220-PE Fab fragments to visualize Kupffer and B cells respectively. Mice were prepared for intravital liver imaging and subsequently treated with 0.4µg GA101.

**Movie S4. Low doses of rituximab fail to induce B cell phagocytosis in the liver**

hCD20Tg mice were injected i.v. with 2µg FITC-conjugated anti-F4/80 Ab and B220-PE Fab fragments to visualize Kupffer and B cells respectively. Mice were prepared for intravital liver imaging and subsequently treated with 0.4µg rituximab.
